# Single-machine scheduling with periodic maintenance and learning effect

**DOI:** 10.1038/s41598-023-36056-w

**Published:** 2023-06-08

**Authors:** Hui Wu, Hongmei Zheng

**Affiliations:** 1 School of Science and Information Science, Qingdao Agricultural University, Qingdao, 266109 China; 2 School of Mathematics and Computer Science, Shaanxi University of Technology, Hanzhong, 723001 China

**Keywords:** Engineering, Mathematics and computing

## Abstract

This paper discusses a single-machine scheduling problem with periodic maintenance activities and position-based learning effect to minimize the makespan. To obtain exact solutions of small-scale problems, one new two-stage binary integer programming model is formulated. In addition, a branch and bound algorithm combining boundary method and pruning rules is also proposed. According to the property of the optimal solution, a special search neighborhood is constructed. A hybrid genetic-tabu search algorithm based on genetic mechanism with tabu technique as an operator is proposed to solve medium-scale and large-scale problems. Moreover, to improve the efficiency of genetic algorithm and hybrid genetic-tabu search algorithm, Taguchi method is used for parameter tuning. Furthermore, computational experiments are carried out to compare the efficiency and performance of these algorithms.

## Introduction

In the traditional machine scheduling, it is common to assume that all machines are always available during the scheduling period. However, such an assumption does not hold true in real process industries and manufacturing systems. In many cases, machines need to be shut down during scheduling due to a variety of reasons, such as preventive maintenance, breakdown repair, etc. Therefore, a realistic scheduling model should consider maintenance activities.

Maintenance activities are divided into corrective maintenance and preventive maintenance^1^. Corrective maintenance is performed after a breakdown has occurred, while preventive maintenance is scheduled in advance. Compared with preventive maintenance, corrective maintenance costs more and causes more serious consequences. Therefore, it is vital to avoid corrective maintenance. The implementation of preventive maintenance activities can effectively decrease the probability of breakdown. In other words, preventative maintenance can improve the availability of machines.

Preventive maintenance activities include replacement of machine parts, inspection, cleaning, lubrication, refueling and so on. In the last thirty years, there are many researchers discussing preventive maintenance in the scheduling literature. Lee and Liman studied the single-machine scheduling constrained by scheduled maintenance to minimize the total flow time, and elucidated that the problem is Non-deterministic Polynomial-time complete (NP-complete)^2^. Lee discussed an availability constraint in parallel machine and single-machine scheduling problems with various performance measures^3^. Subsequently, Qi et al. extended the above problem to consider machine maintenance activities and job scheduling at the same time^4^. Furthermore, Qi researched the single-machine scheduling problem under multi-maintenance and analyzed the performance of heuristic algorithms^5^. Zammori et al. focused on the single-machine scheduling with sequence dependent setup times and maintenance tasks simultaneously^6^. Chen et al. addressed the single machine scheduling problem derived from a rotor production workshop in which preventive maintenance with different improvement effectiveness^7^.

Recently, the single-machine scheduling problems considering preventive maintenance activities and makespan criteria have received considerable attention. Ji et al. studied the problem with nonresumable jobs and analyzed the worst-case ratio of the longest processing time algorithm^8^. Wong et al. employed genetic algorithm (GA) to minimize the makespan^9^. Ángel-Bello et al.^10,11^ and Pacheco et al.^12,13^ developed different heuristic approaches and intelligence algorithms to solve the problem with preventive maintenance and sequence-dependent set-up times. More recent research results in this regard can also be found in^14–18^.

In deterministic scheduling circumstances, it is usually assumed that the processing times of jobs are known and fixed during the scheduling period. However, in many real manufacturing systems, workers will accumulate experience and improve skills after repeatedly handling the same or similar tasks, resulting in a reduction in actual processing time. This phenomenon is called learning effect. Biskup^19^ and Cheng and Wang^20^ independently applied the learning effect to single-machine scheduling for the first time. Biskup demonstrated that the single-machine scheduling with position-based learning effect is solvable in polynomial time when the total flowtime or the deviation from the common due date is minimized^19^. Mosheiov and Sidney presented a general position-based learning effect model by extending the Biskup model, and analyzed the complexity of scheduling problems with various criteria in various machine environments^21^. Wu and Lee further extended the learning effect model, in which the actual processing time is related to its position and the total processing time of the currently processed jobs^22^. Afterwards, Biskup made a comprehensive review on scheduling with learning effects^23^. More recent researches on single-machine scheduling with learning effects, see^24–26^.

Although more and more studies have been done on the single-machine scheduling with preventive maintenance or learning effect, few researchers have considered preventive maintenance activities and learning effect simultaneously. Vahedi-Nouri et al. studied the single-machine scheduling with position-based learning effect and multiple availability constraints to minimize the total completion time^28^. They developed a binary integer programming (BIP) model and proposed a branch and bound (B&B) algorithm, simulated annealing (SA) algorithm and GA to solve the problem. Subsequently, Vahedi-Nouri et al. combined learning effect with flexible maintenance activities to study the non-permutation flow shop scheduling^29^. The sum of maintenance costs and tardiness costs is minimized by determining the completion time of maintenance activities and the sequence of jobs. Bai et al. investigated the single-machine scheduling with an availability constraint and DeJong's learning effect, and proposed a fully polynomial-time approximation scheme^30^.

Periodic maintenance is a common type of preventive maintenance. Machines must be maintained after a fixed period of time. In this study, we consider a single-machine scheduling with periodic maintenance and Biskup’s learning effect simultaneously. The criterion is to minimize the makespan. To the current author's knowledge, the scheduling problem has not been studied. In order to solve the problem, one new two-stage BIP model is developed, and a B&B algorithm combining boundary method and some dominance properties are also proposed. To obtain the near-optimal solutions of medium-scale and large-scale problems, GA is introduced. Additionally, a special neighborhood is constructed according to the dominance property of the scheduling problem. Subsequently a hybrid genetic-tabu search algorithm (HGTSA) based on GA and tabu search (TS) with the special neighborhood is presented. Also, to improve the performance of GA and HGTSA, Taguchi method is used for parameter tuning. Furthermore, computational experiments are performed to analyze and evaluate the performance and efficiency of the proposed algorithms. Computational results verified that the two-stage BIP model is powerful enough. Moreover, the computational results also demonstrated that HGTSA, combined the advantages of GA and TS, has strong global search ability and powerful local search ability.

The rest of this study is organized as follows. The problem description and notation are presented in the following section. In “Properties” section, one new two-stage BIP model is developed. In “Two-stage BIP model”, some dominance properties of the considered problem are obtained. In “Proposed algorithms” section, three algorithms including B&B algorithm, GA and HGTSA are presented. In “Computational experiments” section, the method of generating test instances is introduced at first, then Taguchi method is employed to tune the parameters of GA and HGTSA, and finally the proposed algorithms are analyzed and evaluated based on the computational results. Conclusions are presented in the last Section of this study.

## Problem description

In this study, we consider a single-machine scheduling with periodic maintenance activities and learning effect simultaneously. The objective is to minimize the makespan. The details of the problem are as follows. There are $n$ nonresumable jobs $J = \{ J_{1} ,J_{2} , \ldots ,J_{n} \}$ to be processed on a single machine. Nonresumable job means that if the process of a job could not be completed before the next maintenance activity, then it must be restarted after maintenance activity. All jobs are independent of each other and available at time zero. Each job $J_{i}$ has a normal processing time $p_{i}$. However, due to the learning effect, the actual processing time $p_{ir}$ of $J_{i}$ is less than or equal to $p_{i}$, which is related to its position $r$ in schedule, refer to^19^. The actual processing time $p_{ir}$ is given by the following formula.

1 $$p_{ir} = p_{i} r^{a} ,i,r = 1,2, \ldots ,n(a \le 0\,{\text{is}}\,{\text{a}}\,{\text{constant}}\,{\text{learning}}\,{\text{index}}).$$

The machine must be shut down for preventive maintenance after a period interval $T$. The time required to perform each maintenance is $t$. The machine cannot handle any job during maintenance.

**Figure 1:**
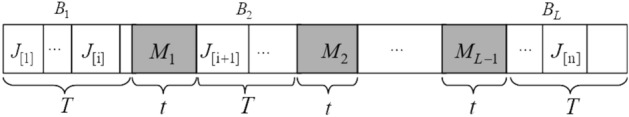
Gantt chart of the problem with periodic maintenance.

In a schedule, jobs processed continuously form a batch, represented by $B$. Therefore, a feasible schedule can be denoted as a series of batches. The total actual processing time of jobs in each batch cannot exceed $T$, and there is a periodic maintenance after a fixed period $T$. The Gantt chart of the current problem is shown in Fig. 1, where $M_{l}$ is the $l$th maintenance and $B_{l}$ is the $l$th batch. $L$ represents the number of batches required to process $n$ jobs. $I_{l}$ denotes the machine idle time between the finishing of the last job in $B_{l}$ and the beginning of $M_{l}$. We suppose that there are $n_{l}$ jobs in batch $B_{l}$. For convenience, let $J_{[i]}$ be the job at the $i$th position in the schedule. Let $p_{[i]}$ and $C_{[i]}$ denote the actual processing time and completion time of $J_{[i]}$, respectively. Let $C_{i}$ denote the completion time of $J_{i}$.

According to the three-field representation proposed by Graham et al.^31^, the current problem can be expressed as $1|pm,nr - le|C_{\max }$, where 1 represents a single machine, $pm$ represents the constraint with periodic maintenance, $nr$ represents the constraint of nonresumable jobs, $le$ represents the jobs with learning effect, and $C_{\max }$ represents that the objective function is makespan.

## Properties

This section will give some properties of the problem $1|pm,nr - le|C_{\max }$.

### Property 1

The problem $1|pm,nr - le|C_{\max }$ is Non-deterministic Polynomial-time hard (NP-hard) in the strong sense.

### Proof

 Problem $1|pm,nr|C_{\max }$ has nonresumable jobs and periodic maintenance, and its criterion is to minimize the makespan. Lee has concluded that problem $1|pm,nr|C_{\max }$ is NP-hard in the strong sense via the 3-Partition problem^3^.

Obviously, the considered problem $1|pm,nr - le|C_{\max }$ with learning effect is also strongly NP-hard. □

### Property 2

In the problem $1|pm,nr - le|C_{\max }$, there exists an optimal schedule in which the jobs in the same batch are arranged in non-decreasing order of their normal processing times.

### Proof

 (by contradiction) Suppose $\pi$ is an optimal schedule, in which there exists two adjacent jobs $J_{i} ,\;J_{j} \in B_{h}$, $p_{i} > p_{j}$, and job $J_{i}$ sequenced precede job $J_{j}$, that is, $\pi$ does not satisfy this conclusion.

Consider the feasible schedule $$\pi^{\prime}$$ derived from $$\pi$$ by exchanging the positions of job $$J_{i}$$ and job $$J_{j}$$. Obviously, with the exception of $$J_{i}$$ and $$J_{j}$$ in $$B_{h}$$, the actual processing times of other jobs in $$\pi$$ will not be affected. For convenience, we assume that the position of $$J_{i}$$ in $$\pi$$ is $$r$$, then the position of $$J_{j}$$ in $$\pi$$ is $$r + 1$$. In optimal schedule $$\pi$$, the sum of the actual processing times of $$J_{i}$$ and $$J_{j}$$ is equal to $$p_{i} r^{a} + p_{j} (r + 1)^{a}$$. In schedule $$\pi^{\prime}$$, the sum of the actual processing times of $$J_{i}$$ and $$J_{j}$$ is equal to $$p_{j} r^{a} + p_{i} (r + 1)^{a}$$. $$p_{i} r^{a} + p_{j} (r + 1)^{a} \le$$$$p_{j} r^{a} + p_{i} (r + 1)^{a}$$. Therefore, $$\left( {p_{i} - p_{j} } \right)r^{a} \le \left( {p_{i} - p_{j} } \right)(r + 1)^{a}$$ and $$p_{i} > p_{j}$$, thus $$r^{a} \le (r + 1)^{a} ,\;\;(a < 0)$$. This is a contradictory inequality. Therefore $$\pi$$ is not an optimal schedule.

Repeat this operation until the jobs in the same batch are in non-decreasing order of their normal processing times, and the theorem can be confirmed. □

Note that when the single machine is continuously available, Problem $1|pm,nr - le|C_{\max }$ will degenerate into the general makespan problem with learning effect and the property still holds true.

Although Property 2 gives the optimal sequence within each batch, idle times should be inserted in the optimal sequence to obtain the optimal schedule.

### Property 3

In the problem $1|pm,nr - le|C_{\max }$, there exists an optimal schedule in which $I_{l} < p_{i} (n_{1} + n_{2} + \cdots + n_{l} { + }1)^{a}$ ($l = 1,2, \ldots ,L - 1$), for all $J_{i} \in B_{h}$, $h = l + 1, \ldots ,L$.

### Proof

(by contradiction) Suppose $\pi$ is an optimal schedule, in which there exists a job $J_{i} \in B_{h}$ such that $I_{l} \ge p_{i} (n_{1} + n_{2} + \cdots + n_{l} )^{a}$ and $h > l$, that is, $\pi$ does not satisfy this conclusion.

Consider the feasible schedule $\pi^{\prime}$ derived from $\pi$ by deleting job $J_{i}$ from its original position and then inserting it into the end of $B_{l}$. Obviously, with the exception of job $J_{i}$ and the jobs sequenced precede job $J_{i}$ in $B_{s}$($s = l + 1, \ldots ,h$), the actual processing times of other jobs in $\pi$ will not be affected. The completion time of job $J_{i}$ will be reduced by at least $t$ in $\pi^{\prime}$. The actual processing times of the jobs scheduled earlier than $J_{i}$ in $B_{s}$($s = l + 1, \ldots ,h$) will be reduced due to the learning effect, and the corresponding completion times will be reduced. The completion times of the jobs scheduled later than $J_{i}$ in $B_{h}$ will be reduced due to the removing of job $J_{i}$ from its original position and the reducing of the actual processing times of the jobs scheduled before $J_{i}$ in the same batch.

Therefore the completion time of the job in schedule $\pi^{\prime}$ is less than or equal to the completion time of the corresponding job in schedule $\pi$. Repeat this operation yields the theorem.□

### Property 4

For the problem $1|pm,nr - le|C_{\max }$, there are two partial schedules $\pi_{1}$ and $\pi_{2}$, which are composed of the same jobs. If the makespan of $\pi_{1}$ is smaller than $\pi_{2}$, then $\pi_{2}$ is dominated by $\pi_{1}$.

### Proof

Let $\pi_{3}$ be the partial schedule composed of the remaining jobs. Since the partial schedule $\pi_{1}$ has the smaller makespan than $\pi_{2}$, the makespan of the full schedule $\left( {\pi_{1} ,\pi_{3} } \right)$ is not greater than that of $\left( {\pi_{2} ,\pi_{3} } \right)$. Thus, $\pi_{2}$ is dominated by $\pi_{1}$. □

The dominance rules given in Property 2–4 can eliminate some partial sequences and reduce unnecessary searches.

## Two-stage BIP model

To obtain the optimal schedule, the actual processing times, the periodic maintenance times and idle times must be considered. The sum of periodic maintenance times is $(L - 1)t$, while the sum of idle times is $\sum\nolimits_{i = 1}^{L - 1} {I_{i} }$. The makespan of $1|pm,nr - le|C_{\max }$ can be expressed as

2 $$C_{\max } = \sum\nolimits_{r = 1}^{n} {\sum\nolimits_{i = 1}^{n} {p_{ir} } } + \sum\nolimits_{i = 1}^{L - 1} {I_{i} } + (L - 1)t = \sum\nolimits_{i = 1}^{n} {p_{[i]} } + \sum\nolimits_{i = 1}^{L - 1} {I_{i} } + (L - 1)t$$

Therefore, the sum of the total actual processing time and the total idle time as well as the number of batches must be minimized in the optimal schedule. We develop a two-stage BIP model to drive the optimal schedule. The BIP model in the first stage is to ascertain the minimum number of batches needed to process $n$ jobs. The second stage is to minimize the sum of the total machine idle time and the total actual processing time. Once the minimum sum is determined in the second stage, the optimal solution can be obtained correspondingly.

Regarding the fact that the jobs in each batch cannot be predetermined, the maximum possible number of batches is taken as the initial condition. Therefore, if $n$ jobs are to be processed, there are at most $n$ batches. However, after sequencing the jobs in combination with the objective function, there will be batches assigned multiple jobs, which will result in some batches without assigned jobs. Then these empty batches will be omitted in our programming model. Recognizing this relation, we define two binary decision variables $x_{ijl}$ and $y_{l}$ in our model.

$$x_{irl} = \left\{ \begin{gathered}1,\quad \, J_{i} \,{\text{is}}\,{\text{sequenced}}\,{\text{in}}\,B_{l} \,{\text{and}}\,{\text{position}}\,r \hfill \\\, 0,\quad \,{\text{otherwise}} \quad\quad\quad\quad\quad\quad\quad\quad\quad\,\,\,\quad\hfill \\ \end{gathered} \right.,$$

$$y_{l} = \left\{ \begin{gathered} 1,\quad n_{l} > 0 \hfill \\ 0,\quad n_{l} = 0 \hfill \\ \end{gathered} \right..$$

The first-stage BIP model is as follows:

3 $${\text{Minimize}}\;L = \sum\limits_{l = 1}^{n} {y_{l} }$$

4 $${\text{Subject}}\,{\text{to}}\sum\limits_{i = 1}^{n} {\sum\limits_{l = 1}^{n} {x_{irl} } = 1} ,\quad r = 1,2, \ldots ,n\;$$

5 $$\sum\limits_{r = 1}^{n} {\sum\limits_{l = 1}^{n} {x_{irl} = 1} } ,\quad i = 1,2, \ldots ,n$$

6 $$\sum\limits_{i = 1}^{n} {\sum\limits_{r = 1}^{n} {x_{irl} } p_{i} r^{a} \le T} ,\quad l = 1,2, \ldots ,n$$

7 $$n_{l} = \sum\limits_{i = 1}^{n} {\sum\limits_{r = 1}^{n} {x_{irl} } ,\quad } l = 1,2, \ldots ,n$$

8 $$n_{l} \cdot M \ge n_{l + 1} ,\quad l = 1,2, \ldots ,n - 1\;$$

9 $$y_{l} \,{\text{is}}\,{\text{binary}},\quad l = 1,2, \ldots ,n$$

10 $$x_{irl} \,{\text{is}}\,{\text{binary}},\quad i = 1,2, \ldots ,n,\quad r = 1,2, \ldots ,n,\quad l = 1,2, \ldots ,n$$

Equation (3) describes the objective of the BIP model in the first stage, that is, to minimize the number of batches needed to process $n$ jobs. Constraints (4) guarantee that there is only one job at each position. Constraints (5) guarantee that each job is assigned to only one position. Constraints (6) ensure that the total actual processing time of jobs in each batch must not exceed the allowable maximum time $T$. Constraints (7) calculate the number of jobs in each batch. In constraints (8), $M$ is a very large positive number. Constraints (8) guarantee that if there are no jobs in batch $B_{l}$, then there are no jobs in batch $B_{l + 1}$. Constraints (9) and (10) set the restriction that decision variables $y_{l}$ and $x_{ijl}$ are binary variables, respectively.

The second-stage BIP model is as follows:

11 $${\text{Minimize}}\,C_{[n]}$$

12 $${\text{Subject}}\,{\text{to}}\sum\limits_{i = 1}^{n} {\sum\limits_{l = 1}^{L} {x_{irl} } = 1} ,\quad r = 1,2, \ldots ,n$$

13 $$\sum\limits_{r = 1}^{n} {\sum\limits_{l = 1}^{L} {x_{irl} = 1} } ,\quad i = 1,2, \ldots ,n$$

14 $$\sum\limits_{i = 1}^{n} {\sum\limits_{r = 1}^{n} {x_{irl} } p_{i} r^{a} \le T} ,\quad l = 1,2, \ldots ,L$$

15 $$n_{l} = \sum\limits_{i = 1}^{n} {\sum\limits_{r = 1}^{n} {x_{irl} } ,\quad } l = 1,2, \ldots ,L$$

16 $$\,n_{l} \cdot M \ge n_{l + 1} ,\quad l = 1,2, \ldots ,L - 1\quad$$

17 $$\begin{aligned} C_{[r]} & = \max \left\{ {C_{[r - 1]} + \sum\limits_{i = 1}^{n} {\sum\limits_{l = 1}^{L} {x_{irl} \cdot p_{i} \cdot r^{a} } ,\quad \sum\limits_{i = 1}^{n} {\sum\limits_{l = 1}^{L} {x_{irl} \cdot [p_{i} \cdot r^{a} + (l - 1) \cdot (T + t)]} } } } \right\}, & r= 1,2, \ldots ,n \\ \end{aligned}$$

18 $$C_{[0]} = 0$$

19 $$x_{irl} \,{\text{is}}\,{\text{binary}},\quad i = 1,2, \ldots ,n,\quad r = 1,2, \ldots ,n,\quad l = 1,2, \ldots ,L$$

Equation (11) describes the objective of the BIP model in the second stage, that is, to minimize the makespan. Constraints (12)–(16) have the same meaning as Constraints (4)–(8). Constraints (17) calculate the completion time of $J_{[r]}$. Constraints (18) and (19) set up the restrictions for $C_{[0]}$ and $x_{ijl}$.

## Proposed algorithms

Property 1 shows that the problem $1|pm,nr - le|C_{\max }$ is NP-hard in the strong sense. In view of the complexity of the problem, three algorithms, namely B&B algorithm, GA and HGTSA, are proposed in this section.

### B&B algorithm

Since the problem $1|pm,nr - le|C_{\max }$ is NP-hard in the strong sense. Implicit enumeration techniques can be used to obtain optimal solutions for small-scale problems. In this subsection, we present a B&B algorithm incorporating with boundary method and several pruning rules. In search tree, each node represents a partial schedule, and each branch represents the addition of a new job to the partial schedule.

#### Upper bound

In the B&B algorithm, enumeration reduction is accomplished by calculating and comparing the upper bounds and the lower bounds. The better the initial upper bound, the more nodes (i.e. partial schedules) we can eliminate in the initial stage of the B&B algorithm, so that the less searching time.

Index the jobs in shortest normal processing time order: $p_{1} \le p_{2} \le \cdots \le p_{n}$. The procedure for solving the initial upper bound is as follows.

Step 1: Arrange the jobs in $\Lambda$-sharp order of their normal processing times,
  $$p_{1} ,p_{3} , \ldots ,p_{n - 1} ,p_{n} ,p_{n - 2} , \ldots ,p_{4} ,p_{2}$$
Step 2: Create the first batch and put the first job in the sequence into the batch.
Step 3: Construct a candidate batch set. A job can be placed in a certain batch only if the cumulative actual processing time of the jobs (including the current job) assigned to the certain batch so far does not exceed time $T$.
Step 4: (1) If the candidate set is empty, create a new batch and assign the current job to the new batch; otherwise, (2) Select a batch from the candidate set such that the difference between $T$ and the cumulative actual processing time of the jobs (including the current job) assigned to the batch so far is the smallest.
Step 5: Repeat Step 3 and Step 4 until all jobs are sequenced.
Step 6: Calculate the makespan of the obtained sequence.

#### Lower bound

At any given node $D$, $\{ J_{1} ,J_{2} , \ldots ,J_{n} \}$ are divided into two categories: jobs scheduled and jobs unscheduled. At node $D$, assume that $n_{D}$ jobs have been scheduled and assigned to positions 1 to $n_{D}$. Let $S_{D} = (J_{[1]} ,J_{[2]} , \ldots ,J_{{[n_{D} ]}} )$ be the partial schedule composed of $n_{D}$ scheduled jobs, and $US_{D}$ be the set of $n - n_{D}$ unscheduled jobs. Let $tt$ denote the total actual processing time of jobs in the last batch in $S_{D}$. Let $z_{L} (D)$ be the lower bound of node $D$, $z_{1} (D)$ be the makespan of $n_{D}$ scheduled jobs, and $z_{2} (D)$ be the makespan of $n - n_{D}$ unscheduled jobs. $z_{1} (D)$ can be obtained directly,

20 $$z_{1} (D) = \mathop {\max }\limits_{{1 \le i \le n_{D} }} C_{[i]} = C_{{[n_{D} ]}} .$$

$z_{2} (D)$ needs to be estimated. Suppose $\pi^{\prime}$ is a partial schedule corresponding to the set $US_{D}$, then $\pi^{\prime}$ starts at the position $n_{D} + 1$. Here batch $B_{i}^{\prime }$ is indexed from the beginning of $\pi^{\prime}$. To obtain a lower bound of node $D$, it is sufficient to ascertain a lower bound of $\pi^{\prime}$. $z_{2} (D)$ is the sum of the total actual processing time, total maintenance time and total idle time corresponding to $n - n_{D}$ unscheduled jobs. Mosheiov had proved that the minimum makespan of the single-machine scheduling with learning effect can be obtained by shortest processing time rule when there is no preventive maintenance^32^. Accordingly, the total actual processing time is given as follows

$$p_{1}^{\prime } \cdot (n_{D} + 1)^{a} + p_{2}^{\prime } \cdot (n_{D} + 2)^{a} + \cdots + p_{{n - n_{D} }}^{\prime } \cdot n^{a} ,$$

where $p_{1}^{\prime } ,p_{2}^{\prime } , \ldots ,p_{{n - n_{D} }}^{\prime }$ are indexed in the non-decreasing order of the normal processing times of jobs in set $US_{D}$. In addition to this, the makespan of $n - n_{D}$ unscheduled jobs is minimum when there is no idle time. Therefore, the needed maintaining time is given as follows

$$\left\lfloor {{{\left[ {tt + p_{1}^{\prime } \cdot (n_{D} + 1)^{a} + p_{2}^{\prime } \cdot (n_{D} + 2)^{a} + \cdots + p_{{n - n_{D} }}^{\prime } \cdot n^{a} } \right]} \mathord{\left/ {\vphantom {{\left[ {tt + p_{1}^{\prime } \cdot (n_{D} + 1)^{a} + p_{2}^{\prime } \cdot (n_{D} + 2)^{a} + \cdots + p_{{n - n_{D} }}^{\prime } \cdot n^{a} } \right]} T}} \right. \kern-0pt} T}} \right\rfloor \cdot t.$$

Then

21 $$\begin{aligned} z_{2} (D) & \ge p_{1}^{\prime } \cdot (n_{D} + 1)^{a} + p_{2}^{\prime } \cdot (n_{D} + 2)^{a} + \cdots + p_{{n - n_{D} }}^{\prime } \cdot n^{a} \\ & \quad + \left\lfloor {{{\left[ {tt + p_{1}^{\prime } \cdot (n_{D} + 1)^{a} + p_{2}^{\prime } \cdot (n_{D} + 2)^{a} + \cdots + p_{{n - n_{D} }}^{\prime } \cdot n^{a} } \right]} \mathord{\left/ {\vphantom {{\left[ {tt + p_{1}^{\prime } \cdot (n_{D} + 1)^{a} + p_{2}^{\prime } \cdot (n_{D} + 2)^{a} + \cdots + p_{{n - n_{D} }}^{\prime } \cdot n^{a} } \right]} T}} \right. \kern-0pt} T}} \right\rfloor \cdot t. \\ \end{aligned}$$

Consequently, we have a lower bound of node $D$ as follows

22 $$\begin{aligned} z_{L} (D) & = C_{{[n_{D} ]}} + p_{1}^{\prime } \cdot (n_{D} + 1)^{a} + p_{2}^{\prime } \cdot (n_{D} + 2)^{a} + \cdots + p_{{n - n_{D} }}^{\prime } \cdot n^{a} \\ & \quad + \left\lfloor {{{\left[ {tt + p_{1}^{\prime } \cdot (n_{D} + 1)^{a} + p_{2}^{\prime } \cdot (n_{D} + 2)^{a} + \cdots + p_{{n - n_{D} }}^{\prime } \cdot n^{a} } \right]} \mathord{\left/ {\vphantom {{\left[ {tt + p_{1}^{\prime } \cdot (n_{D} + 1)^{a} + p_{2}^{\prime } \cdot (n_{D} + 2)^{a} + \cdots + p_{{n - n_{D} }}^{\prime } \cdot n^{a} } \right]} T}} \right. \kern-0pt} T}} \right\rfloor \cdot t. \\ \end{aligned}$$

#### Pruning rules

Pruning rules can reduce unnecessary searches, which can greatly improve the search speed and the computational efficiency. In this subsection, we will propose three pruning rules.

*Rule 1* If the lower bound corresponding to node $D$ is greater than the current upper bound, then $D$ should be deleted.

For any job $J_{i} \in US_{D}$, has normal processing time $p_{i}$. A new node $D_{i}$ can be obtained from node $D$ by attaching $J_{i}$ at the end of $S_{D}$, i.e. at the position of $n_{D} + 1$.

*Rule 2* In the current last batch $tt + p_{i} \cdot (n_{D} + 1)^{a} \le T$, if $p_{i} \cdot n_{D}^{a} < p_{{[n_{D} ]}}$, then $D_{i}$ should be eliminated.

Rule 2 is based on the Property 2 of the optimal schedule. According to Property 2, the jobs in the same batch should be arranged in non-decreasing order of their normal processing times.

*Rule 3* In the current last batch $tt + p_{i} \cdot (n_{D} + 1)^{a} > T$, if $tt + p_{\min } \cdot (n_{D} + 1)^{a} \le T$, where $p_{\min } = \mathop {\min }\limits_{{J_{j} \in US_{D} }} \{ p_{j} \}$, then $D_{i}$ should be eliminated.

For Rule 3, job $J_{i}$ will result in a new batch. Condition follows Property 3.

#### Algorithm steps

In B&B algorithm, there are many nodes in search tree to be selected. We use depth-first strategy by which we can avoid too many nodes to be saved in computer memory. The detailed steps of the B&B algorithm are described as follows.

Step 1: Use the makespan obtained by the method proposed in “Upper bound” as the initial upper bound of the B&B algorithm.
Step 2: Initialize root node $D$, such that $S_{D} = \Phi$, $US_{D} = \{ J_{1} ,J_{2} , \ldots ,J_{n} \}$, $n_{D} = 0$, $tt = 0$.
Step 3: If the search tree is already empty, then stop. Otherwise, select an unsearched node $D$.
Step 4: If $D$ is a leaf node, that is, $US_{D} = \Phi$, then a complete schedule has been obtained. According to formula (2), calculate the makespan. If it is less than the current upper bound, use it to update the current upper bound. Otherwise, eliminate the leaf node. Go to Step 3.
Step 5: For any job $J_{i} \in US_{D}$, new node $D_{i}$ can be obtained from node $D$ by attaching $J_{i}$ at the end of $S_{D}$.
Step 6: In the case of $tt + p_{i} \cdot (n_{D} + 1)^{a} \le T$, if the new node $D_{i}$ satisfies Rule 2, eliminate it. Otherwise, calculate the lower bound corresponding to $D_{i}$ according to formula (22). If the lower bound satisfies Rule 1, eliminate it. Otherwise, put the new node $D_{i}$ in the search tree. Let $tt = tt + p_{i} \cdot (n_{{_{D} }} + 1)^{a}$, $n_{D} = n_{D} + 1$. Go to Step 3.
Step 7: In the case of $tt + p_{i} \cdot (n_{D} + 1)^{a} > T$, if the new node $D_{i}$ satisfies Rule 3, eliminate it. Go to Step 3.
Step 8: In the case of $tt + p_{i} \cdot (n_{D} + 1)^{a} > T$ for any $J_{i} \in US_{D}$, assigning job $J_{i}$ into the next new batch, new node $D_{i}$ can be obtained. Calculate the lower bound corresponding to $D_{i}$ according to formula (22). If the lower bound satisfies Rule 1, eliminate it. Otherwise, put the new node $D_{i}$ in the search tree. Let $tt = p_{i} \cdot (n_{{_{D} }} + 1)^{a}$, $n_{D} = n_{D} + 1$. Go to Step 3.

## GA

GA, first proposed by Holland^33^, is an artificial evolutionary algorithm. One of the main differences between the algorithm and other meta-heuristic algorithms is that it uses a set of solutions rather than a single one. Because of the efficiency of GA in solving discrete optimization problems, some researchers have developed it to solve scheduling problems. The mechanisms of the GA are briefly described as follows:

1. Chromosome structure. In the considered single-machine scheduling problem, each feasible schedule is encoded as a chromosome containing $n$ integers, in which each integer stands for a job. Integer $i$ at the $j$-th position of the chromosome indicates that $J_{i}$ is at the $j$-th position of the scheduling sequence. The chromosome structure indicates the sequence of jobs and their normal processing times.
2. Population initialization. Determining population size is particularly important in the GA. Population size is too small to obtain a good solution, however population size is too large will consume too much CPU time. According to the Taguchi method tuning experiment, the population size is set as $n_{pop} = 200$. In order to enhance search efficiency, $n_{pop}$ best solutions are selected from 2000 randomly generated solutions as the initial population.
3. Fitness function. Fitness function specifies how suitable a solution is. Different candidate solutions could be evaluated according to their fitness values. In the current problem, the fitness function is based on formula (2), that is
  $$z(\pi ) = \sum\nolimits_{r = 1}^{n} {\sum\nolimits_{i = 1}^{n} {p_{ir} } } + \sum\nolimits_{i = 1}^{L - 1} {I_{i} } + (L - 1)t = \sum\nolimits_{i = 1}^{n} {p_{[i]} } + \sum\nolimits_{i = 1}^{L - 1} {I_{i} } + (L - 1)t$$

The fitness function of individual $\pi_{s}$ is the following function

23 $$fitness(\pi_{s} ) = \mathop {\max }\limits_{t = 1}^{{n_{pop} }} (z(\pi_{t} )) - z(\pi_{s} ) + \varepsilon ,$$

where $\varepsilon$ is a positive constant.

(4) Selection strategy. In this research, we select individuals according to fitness values using Roulette Wheel method. All individuals have the opportunity to be selected, but better individuals have higher probability. The probability to select $\pi_{s}$ is
  24 $$P_{{\pi_{s} }} = \frac{{fitness(\pi_{s} )}}{{\sum\limits_{t = 1}^{{n_{pop} }} {fitness(\pi_{t} )} }}.$$
(5) Crossover operator and mutation operator. Crossover operator generates offspring chromosomes by exchanging parts of parent chromosomes from the existing population. Partially-matched crossover method is employed in the current problem. Mutation operator can maintain population diversity to avoid GA falling into local optimum by slightly disturbing of the selected parent chromosomes. The mutation operator used in the stated problem is to randomly select two mutation positions and exchange the jobs at the mutation positions. According to the Taguchi method tuning experiment, the crossover probability is taken as $Pc = 0.85$, and the mutation probability is taken as $Pm = 0.15$.
(6) Regeneration. Random selection cannot guarantee that the best chromosomes will survive to the next generation. To avoid this situation, we use a mixed method to attain next generation. Merge the current population and the offspring chromosomes generated by the crossover operator and mutation operator, sort them according to their fitness values, and select the best $n_{pop}$ chromosomes as the next generation.
(7) Termination. Stopping criteria is the maximum number of iterations. According to the Taguchi method tuning experiment, the maximum iteration number is set to $INGA = 250$.

## HGTSA

### TS

**Figure 2:**
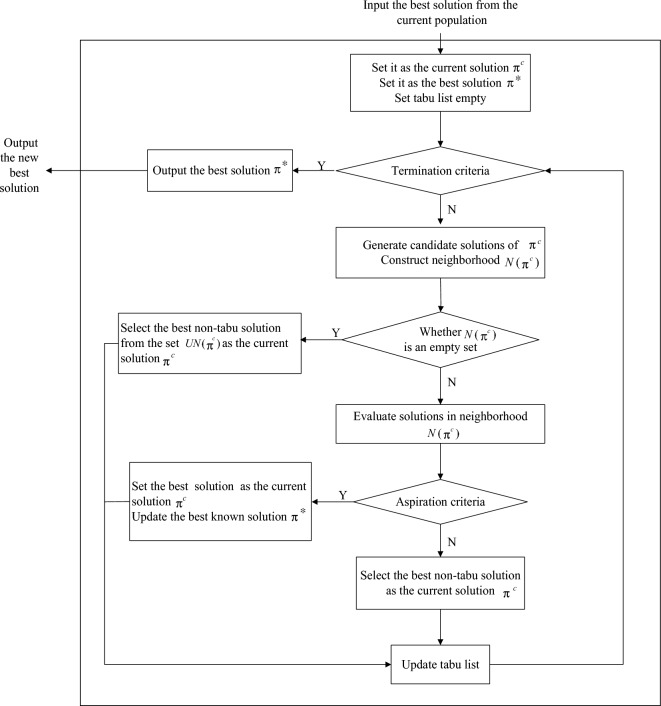
The basic workflow of TS.

TS algorithm, originally presented by Glover in 1986^34^, is an iterative improvement algorithm. To avoid falling into local optimum, TS allows exploration of solution spaces that do not necessarily optimize the objective function value. The main mechanisms of TS algorithm consist of initial solution, neighborhood, tabu list, aspiration criterion and terminate criterion. The basic framework is shown in Fig. 2.

1. Initial solution. Initial feasible solution is used to generate neighborhood for the next searching process. A better initial solution can increase the convergence rate and reduce the searching time.
2. Neighborhood. A pairwise swap method is used to construct neighborhood structure. It is one of the most commonly used methods and easy to implement. For the current schedule $\pi$, candidate solutions are obtained by performing pairwise swaps on $\pi$. Consequently, the maximum number of candidate solutions corresponding to $\pi$ is $\sum\nolimits_{i = 1}^{n - 1} {(n - i)}$. The number of candidate solutions will increase significantly as the scale of the problem increases. Due to the large scale of candidate solutions and the resulting great computational burden, a constraint must be made to restrict the size of the neighborhood without affecting the efficiency of the algorithm. Better candidate solutions can accelerate the convergence speed. Motivated by this, the current problem uses the dominance Property 4 as the constraint. The neighborhood is denoted by $N(\pi )$. We put the candidate solutions whose makespan is no more than $C_{\max } (\pi )$ in $N(\pi )$, and the others in another set, denoted by $UN(\pi )$. For instance, we swap $J_{i}$ and $J_{j}$ in the current schedule $\pi = (S_{1} ,J_{i} ,S_{2} ,J_{j} ,S_{3} )$ to obtain schedule $\pi^{\prime} = (S_{1} ,J_{j} ,S_{2} ,J_{i} ,S_{3} )$ in which $S_{1} ,S_{2}$ and $S_{3}$ denote partial schedules. It is obviously that $(S_{1} ,J_{i} ,S_{2} ,J_{j} )$ and $(S_{1} ,J_{j} ,S_{2} ,J_{i} )$ are composed of the same set of jobs. If $(S_{1} ,J_{i} ,S_{2} ,J_{j} )$ is dominated by $(S_{1} ,J_{j} ,S_{2} ,J_{i} )$, we put $\pi^{\prime}$ in $N(\pi )$. The better the current schedule $\pi$ is, the smaller the corresponding neighborhood scale will be. However, a situation could arise that the neighborhood $N(\pi )$ is an empty set. In such a situation, we select the best non-tabu solution from $UN(\pi )$ as the current solution.
3. Tabu list. To avoid falling into local optimum, tabu list is used to prevent repeated searches for the same solution visited recently within a certain number of iterations. If a pairwise swap is selected, then its inverse swap is forbidden and added to the tabu list. The forbidden swaps in the tabu list reduce the possibility of search loop. The length of the tabu list has a great influence on search speed and quality. Too small the length will lead to frequent repeated searches. Instead, it will rule out the search paths that might generate good solutions. The larger the problem, the longer the length should be. In the current problem, we set the length of the tabu list according to the size of the problem, as follows.
  25 $$TL = \left[ {\frac{n(n - 1)}{2}} \right]^{\frac{1}{2}}$$

where $TL$ and $n$ denote the length of the tabu list and the size of the problem, respectively. Update the tabu list using the First In First Out rule.

(4) Aspiration criterion. Aspiration criterion is the key step for the algorithm to realize global optimization. If there is a neighborhood solution superior to the current solution, whether or not the corresponding swap is in the tabu list, we select it as the current solution, then update the tabu list and the current best solution.
(5) Stopping criterion. In the current problem, set termination conditions according to the objective control principle. If the current best solution is not improved within continuous *MNTS* steps, the algorithm will be terminated. Here, *MNTS* is a given number, and the value of *MNTS* will be tuned for a specific test instance by tradeoff between the solution quality and CPU time.

### Hybrid algorithm

**Figure 3:**
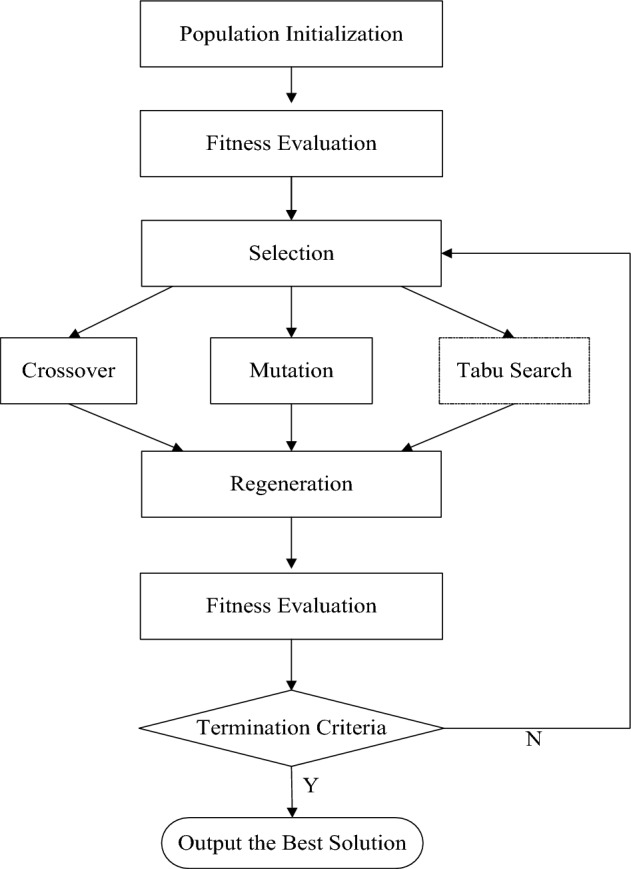
The basic workflow of HGTSA.

TS has capability of fast local search is utilized to execute exploitation, and GA has strong global optimization feature is applied to conduct exploration. The HGTSA uses TS as an operator of GA to improve the performance of local search. HGTSA combines the advantages of population-based and local search methods together. Therefore, HGTSA has powerful searching ability which ensures global optimization and fast convergence rate. The flowchart of the HGTSA framework is shown in Fig. 3. The main steps of the HGTSA are as follows:

Step 1: Set the parameters of the HGTSA; Choose $n_{pop}$ best solutions out of 2000 randomly generated solutions as the initial population.
Step 2: Evaluate the fitness function value of each chromosome.
Step 3: Select parent chromosomes using Roulette Wheel method to execute crossover operation, mutation operation. Select the best solution from the selected parent chromosomes to execute TS.
  Step 3.1: Set the best solution from the selected parent chromosomes as the current solution $\pi^{c}$ of TS.
  Step 3.2: If the termination criterion of TS is satisfied, output the best solution $\pi^{*}$ of TS, and go to Step4; otherwise, go to Step 3.3.
  Step 3.3: Generate candidate solutions according to the pairwise swap rule and construct neighborhood $N(\pi^{c} )$ applying the dominance Property 4. If the neighborhood $N(\pi^{c} )$ is a non-empty set, evaluate the solutions in neighborhood $N(\pi^{c} )$, then go to Step 3.4, otherwise, select the best non-tabu solution from the set $UN(\pi^{c} )$ as the current solution $\pi^{c}$, then go to Step 3.7.
  Step 3.4: If the aspiration criterion of TS is satisfied, go to Step 3.5, otherwise, go to Step 3.6.
  Step 3.5: Select the best solution as the current solution $\pi^{c}$. Update the best known solution $\pi^{*}$ of TS, then go to Step3.7.
  Step 3.6: Select the best solution from non-tabu solutions as the current solution $\pi^{c}$, go to Step3.7.
  Step 3.7: Update the tabu list, then go to Step3.2.
Step 4: Merge the current population and the offspring chromosomes generated by the crossover operator, mutation operator and TS.
Step 5: Evaluate chromosomes according to their fitness values. Choose $n_{pop}$ best solutions as the next generation and update the best known solution.
Step 6: If the termination criterion of GA is satisfied, output the best solution and stop; otherwise, go to Step 3.

## Computational experiments

In the section, numerical experiments are carried out to evaluate the efficiency and accuracy of the proposed algorithms. The two-stage BIP model is resolved by optimizer LINGO. B&B, GA and HGTSA are coded in MATLAB programming software. All computational experiments are performed on a PC with Intel(R) Core(TM) i7- 5500U@ 2.4 GHz 8 GB RAM.

### Generation of test instances

Since there is no benchmark for $1|pm,nr - le|C_{\max }$, all test instances in this paper are generated randomly. The parameters established by Vahedi-Nouri et al.^28^ and Hsu et al.^35^ are adopted, due to the similarity of the problems. Methods of data generation are briefly outlined in Table 1.

**Table 1:** Data generation of test instances.

Parameter	Notation	Value
Problem size	$n$	{5, 8, 10, 15, 20, 25, 30, 35, 100, 200, 500, 1000}
Normal processing time	$p_{i}$	Integer uniform distribution [3,60]
Maintenance duration time	$t$	Normal distribution ($\mu { = }20$,$\delta { = }5$)
Maintenance period	$T$	Normal distribution ($\mu { = }\sum {p_{i} } /3{,}\delta { = 10}$)
Learning index	$a \le 0$	Normal distribution ($\mu { = }0.2$,$\delta { = }0.07$)

The test instances are categorized into three groups: small-size problems with 5, 8, 10, 15, 20, 25, 30, 35 jobs; medium-size problems with 100, 200 jobs, and large-size problems with 500, 1000 jobs. In addition, $T$ cannot be less than the maintenance duration time $t$ and the maximum normal processing time $\mathop {\max }\limits_{1 \le i \le n} \left\{ {p_{i} } \right\}$.

### Taguchi method

Parameters in meta-heuristic algorithm have a major impact on its performance optimization. They should be determined according to specific problems. In this study, Taguchi method is used to tune the parameters in the proposed algorithms. The method, pioneered by Genechi Taguchi in the 1960s, has been widely used for its robustness^29,36^. The Taguchi method, through a set of orthogonal arrays, specifies how to perform the minimum number of experiments to provide full information on relevant parameters^37^. In the Taguchi orthogonal method, the experimental results obtained from a few experiments are converted into signal-to-noise ratio (SNR) to determine the optimal levels of parameters. The SNR for $1|pm,nr - le|C_{\max }$ is calculated by Phadke^38^:

26 $$\eta_{j} = - 10\lg \left( {\frac{1}{N}\sum\limits_{i = 1}^{N} {y_{i}^{2} } } \right),$$

where $N$, $j,\;$ and $y_{i}$ indicate the number of tests, the instance index, and test value respectively. The SNR of each parameter level is obtained by averaging the SNRs of each parameter at the same level.

**Figure 4:**
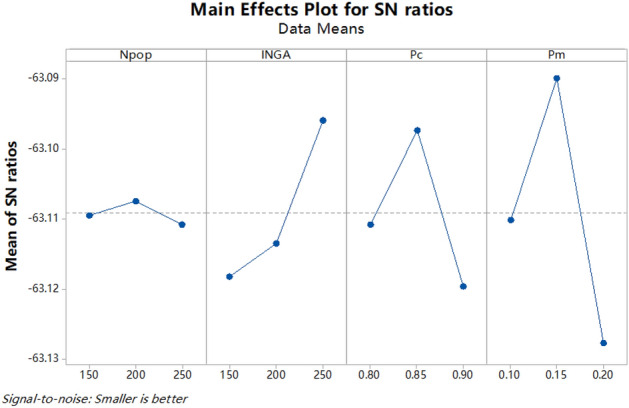
Main effects plot of each parameter.

The population size, maximum number of iterations, mutation probability and crossover probability in GA are tuned by the Taguchi method. The mentioned parameters with their levels are illustrated in Table 2. Since there are four parameters each with three levels, based on Taguchi orthogonal arrays, we perform 9 different experiments ($L_{9} (3^{4} )$), each of which involves a combination of different parameter levels. To make robust results of the proposed algorithms, these experiments are performed with five replications on different test problems. The mean SNR of each parameter level is calculated and its trend is shown in Fig. 4. Since the objective function of $1|pm,nr - le|C_{\max }$ is the smaller the better type, the best level of each parameter is the one with the highest mean SNR. Based on the above analysis, the best level of each parameter is shown in Table 3.

**Table 2:** Parameters and their levels.

Parameter		Level	
	1	2	3
Population size ($n_{pop}$)	150	200	250
Iteration number in GA (INGA)	150	200	250
Crossover probability ($Pc$)	0.8	0.85	0.9
Mutation probability ($Pm$)	0.1	0.15	0.2

**Table 3:** Best levels for the parameters.

Parameter	Best level
Population size ($n_{pop}$)	200
Iteration number in GA (INGA)	250
Crossover probability ($Pc$)	0.85
Mutation probability ($Pm$)	0.15

### Performance comparison of the proposed methods

In preliminary tests, even if the execution time exceeds 3600 s, the two-stage BIP model cannot achieve optimal solutions for several test instances with 25 jobs. In view of this, we set a maximum running time restriction of 3600 s for the proposed methods. Due to the nature of heuristic methods, each instance is solved and run 5 times by the proposed methods respectively. For evaluating the accuracy of the proposed methods, the percentage of error is employed. We calculate the percentage of error according to formula (27)

27 $$error\% = \frac{best\;solution - optimal\;solution}{{optimal\;solution}} \times 100\% ,$$

where *best solution* is the solution obtained by GA or HGTSA, and *optimal solution* is the solution obtained by B&B or two-stage BIP model.

**Figure 5:**
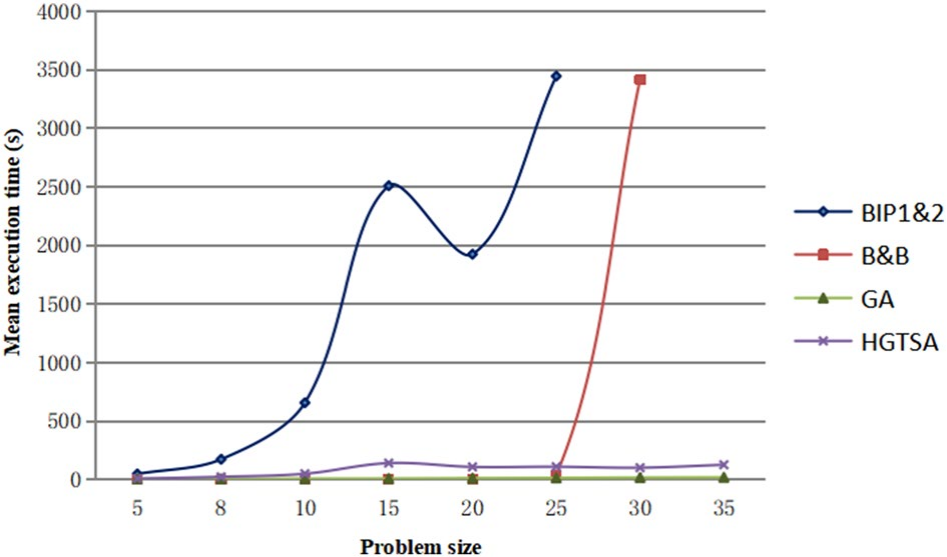
Comparison of the mean execution times of the proposed methods for small-scale instances (5–35 jobs).

**Figure 6:**
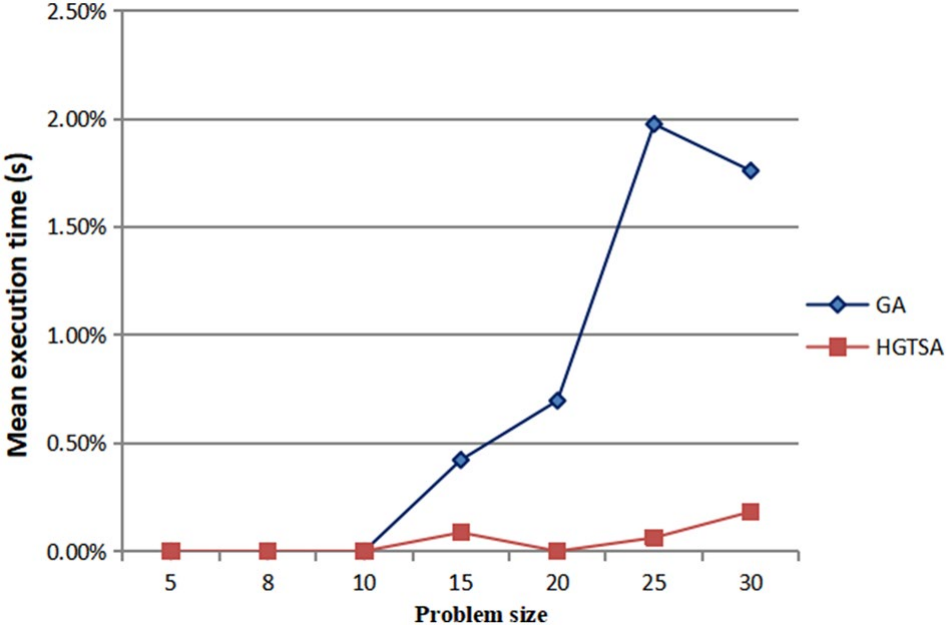
Comparison of the mean percentage errors of HGTSA and GA for small-scale instances (5–30 jobs).

**Figure 7:**
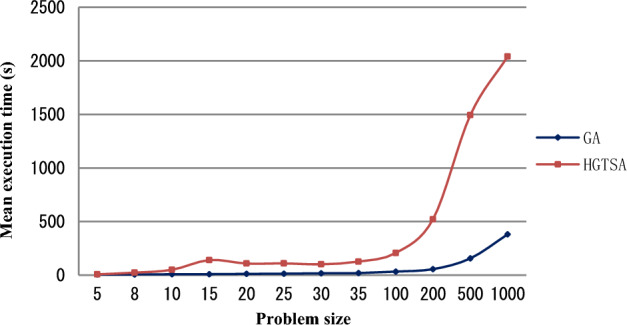
Comparison of the mean execution times of HGTSA and GA for all test instances (5–1000 jobs).

**Figure 8:**
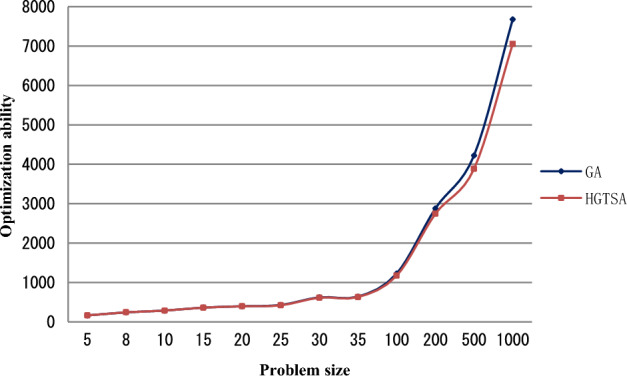
Comparison of the optimization ability of HGTSA and GA for all test instances (5–1000 jobs).

**Figure 9:**
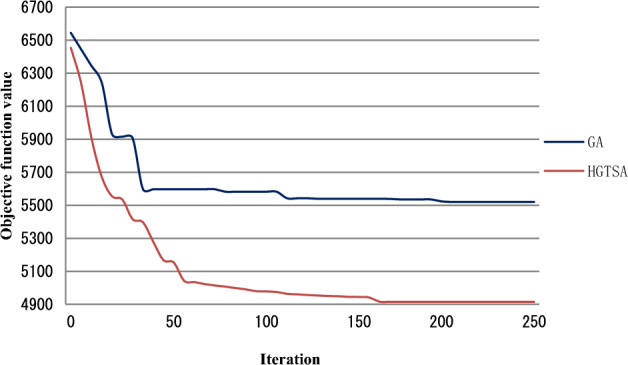
Comparison of the convergence of HGTSA and GA for 1000 jobs with learning index 0.3

The results for solving the test instances are shown in Tables 4 and 5. Columns 1, 2, 3 and 4 describe job size, maintenance period, maintenance duration time and learning index respectively. In Table 4, columns 5–12, 14–15 describe the mean execution time (in seconds) for solving each problem and the best objective values obtained by the two-stage BIP model, B&B, GA and HGTSA respectively. Columns 13 and 16 in Table 4 present the percentages of errors obtained from GA and HGTSA respectively. In Table 5, columns 5–8, 10–11 describe the mean execution time (in seconds) for solving each problem and the best objective values obtained by B&B, GA and HGTSA respectively. Columns 9 and 12 in Table 5 present the percentages of errors obtained from GA and HGTSA respectively. The symbol “–” indicates that the corresponding algorithm cannot solve the problem. In order to more intuitively evaluate the accuracy and efficiency of the proposed methods, Figs. 5, 6, 7, 8 are presented based on Tables 4 and 5. Figure 5 shows the mean execution times of the proposed methods for small-scale instances (5–35 jobs). The mean execution time of the B&B is less than that of the two-stage BIP model, GA and HGTSA for the problem with no more than 20 jobs, but the execution times of GA and HGTSA increase almost linearly and are much less than that of the B&B for the problem with more than or equal to 25 jobs. Although the execution time of the two-stage BIP model increases exponentially with the increase of the problem scale, compared with the general BIP models such as^27^ and^28^, the two-stage BIP model has significantly improved solving ability, which can solve up to 20–25 jobs. The B&B can find optimal solutions for instances with up to 30 jobs in a reasonable running time. Figure 6 illustrates that the percentages of errors of GA and HGTSA for small-scale instances (5–30 jobs) are very small, which indicates that GA and HGTSA have the potential to obtain near-optimal solutions in solving medium and large-scale problems. The mean execution times of GA and HGTSA, for all test instances, are depicted in Fig. 7. Although the mean execution time of HGTSA is longer than that of GA, both algorithms can solve large-scale instances of up to 1000 jobs within the maximum running time restriction. Figure 8 shows the optimization ability of GA and HGTSA in solving all test instances. The best solution obtained by HGTSA is superior to the best solution obtained by GA for the instances with more than 100 jobs. The computational results also demonstrated that HGTSA, combined the advantages of GA and TS, has strong global search ability. Figure 9 presents the convergence of GA and HGTSA for 1000 jobs with learning index 0.3 and demonstrates that the TS with special neighborhood makes HGTSA search deeply and efficiently much more than GA in each step of the iteration. Figures 8 and 9 indicate the optimization ability of HGTSA is better than that of GA for the medium-size problems and large-size problems, which reflects that GA is prone to fall into local optimal, and also reflects that HGTSA based on GA and TS has strong local search ability and powerful global search ability.

**Table 4:** Performance comparison of two-stage BIP method, B&B, GA and HGTSA for test problems.

$n$	$T$	$t$	$a$	BIP-1		BIP-2		B&B		GA			HGTSA		
				Value	Time	Value	Time	Value	Time	Value	Time	Error%	Value	Time	Error%
5	93	16	− 0.1	2	1	172.36	52	172.36	0.15	172.36	5.82	0.000	172.36	8.13	0.000
			− 0.2	2	1	157.10	42	157.10	0.16	157.10	6.34	0.000	157.10	8.09	0.000
8	129	24	− 0.1	2	6	277.19	231	277.19	0.18	277.19	6.82	0.000	277.19	23.99	0.000
			− 0.3	2	5	207.64	105	207.64	0.14	207.64	6.84	0.000	207.64	23.87	0.000
10	125	18	− 0.1	3	18	318.57	523	318.57	0.15	318.57	8.73	0.000	318.57	51.48	0.000
			− 0.2	2	19	252.60	754	252.60	0.18	252.60	8.65	0.000	252.60	49.73	0.000
15	151	24	− 0.1	3	966	409.66	2 480	409.66	0.41	411.95	9.5	0.563	410.37	144.89	0.173
			− 0.2	2	966	312.93	602	312.93	0.29	313.81	9.58	0.281	312.93	137.25	0.000
20	194	19	− 0.2	3	979	451.27	691	451.27	5.38	451.27	13.04	0.000	451.27	122.68	0.000
			− 0.3	2	974	337.39	1200	337.39	6.28	342.09	11.28	1.393	337.39	95.23	0.000
25	252	24	− 0.2	2	1021	470.30	2423	470.30	43.78	478.25	13.55	1.690	470.89	97.88	0.125
			− 0.3	2	931	–	–	368.66	36.55	377.00	15.33	2.262	368.66	123.13	0.000

**Table 5:** Performance comparison of B&B, GA and HGTSA for test problems.

$n$	$T$	$t$	$a$	B&B		GA			HGTSA		
				Value	Time	Value	Time	Error%	Value	Time	Error%
30	287	24	− 0.1	711.20	3 271	720.55	17.61	1.315	711.20	104.87	0.000
			− 0.2	515.43	3 559	526.80	17.67	2.206	517.31	102.54	0.365
35	331	22	− 0.1	–	–	824.93	19.67	–	812.11	116.23	–
			− 0.3	–	–	454.47	19.16	–	446.67	136.99	–
100	676	28	− 0.2	–	–	1 464	33.58	–	1 413	208.75	–
			− 0.3	–	–	989.97	33.65	–	935.33	206.74	–
200	1650	27	− 0.1	–	–	4 070	56.44	–	3 961	526.46	–
			− 0.3	–	–	1 687	56.08	–	1 545	519.98	–
500	3033	28	− 0.2	–	–	5 289	154.53	–	4 954	1 776.80	–
			− 0.3	–	–	3 160	158.20	–	2 812	1 254.95	–
1000	4666	28	− 0.2	–	–	9 836	365.17	–	9 189	1 899.53	–
			− 0.3	–	–	5 520	397.40	–	4 915	2 198.46	–

## Conclusions

In this paper, we investigated a single-machine scheduling with periodic maintenance and learning effect while the makespan is minimized. We presented one new two-stage BIP to model the problem. Additionally, we derived some properties about optimal schedule and applied them to the B&B algorithm. Considering that the problem is NP-Hard in the strong sense, we introduced the GA and HGTSA for medium and large-scale problems. Extensive computational experiments have demonstrated the effectiveness of the proposed methods. The proposed two-stage BIP model is powerful enough to solve up to 20 or 25 jobs. The B&B algorithm can find optimal solutions for problems with up to 30 jobs within the maximum running time restriction. Meanwhile, Taguchi method was used for specifying the optimal parameter level to improve the performance of GA and HGTSA. Computational results showed that the proposed HGTSA, combined the advantages of TS and GA, is a promising and effective algorithm. HGTSA performed better than GA with more accurate solutions and faster convergence rate in solving medium and large-scale problems. For interested researchers, it is suggested to extend the problem to more machines or uncertain environments in the future. In addition, HGTSA can also be combined with other methods to solve the multi-objective problem in the production field.

## Data Availability

The authors confirm that all data generated or analysed during this study are included in this published article.
