# Author Correction: Single-machine scheduling with periodic maintenance and learning effect

**DOI:** 10.1038/s41598-023-39460-4

**Published:** 2023-08-03

**Authors:** Hui Wu, Hongmei Zheng

**Affiliations:** 1 School of Science and Information Science, Qingdao Agricultural University, Qingdao, 266109 China; 2 School of Mathematics and Computer Science, Shaanxi University of Technology, Hanzhong, 723001 China

Correction to:* Scientific Reports* 10.1038/s41598-023-36056-w, published online 08 June 2023

The original version of this Article contained errors in Figures 5 and 6, as well as in the Properties section.

In Figures 5 and 6, the running time of the code was incorrectly presented as negative. The original Figures 5 and 6 and accompanying legends appear below.

**Figure 5:**
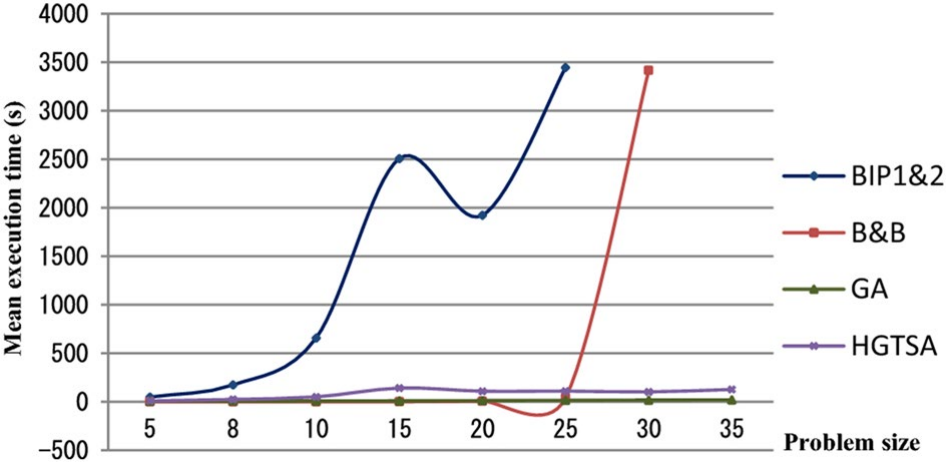
Comparison of the mean execution times of the proposed methods for small-scale instances (5–35 jobs).

**Figure 6:**
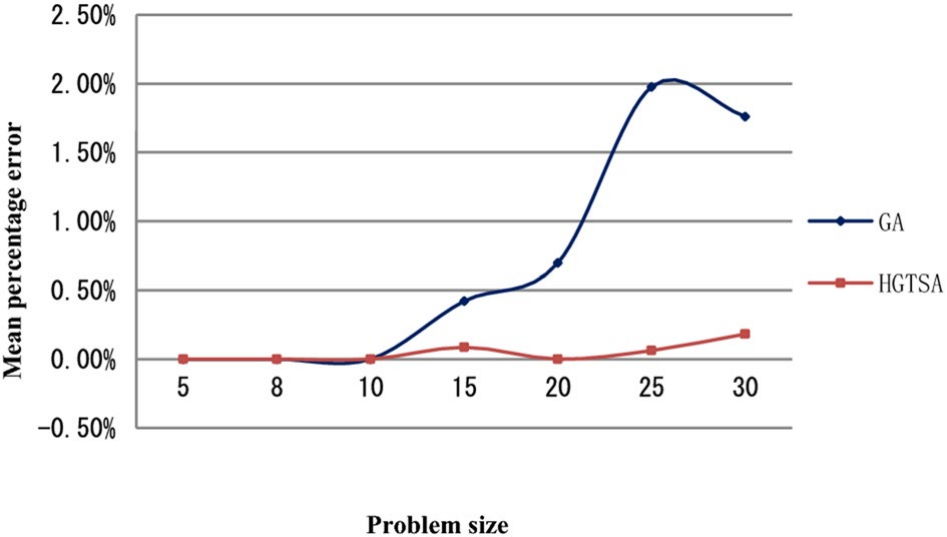
Comparison of the mean percentage errors of HGTSA and GA for small-scale instances (5–30 jobs).

In addition, in the Properties section, under the subheading ‘Property 2’,

“For convenience, we assume that the position of ${J}_{i}$ in $\pi$ is $r$, then the position of ${J}_{i}$ in $\pi$ is $r+1$.”

now reads:

“For convenience, we assume that the position of ${J}_{i}$ in $\pi$ is $r$, then the position of ${J}_{j}$ in $\pi$ is $r+1$.”

The original Article has been corrected.

